# RNA-binding protein AUF1 suppresses miR-122 biogenesis by down-regulating Dicer1 in hepatocellular carcinoma

**DOI:** 10.18632/oncotarget.24079

**Published:** 2018-01-09

**Authors:** Xia Wu, Yingzhuo Yang, Yike Huang, Yang Chen, Tianying Wang, Shuo Wu, Lei Tong, Yan Wang, Lexun Lin, Meili Hao, Zhao-Hua Zhong, Fengmin Zhang, Wenran Zhao

**Affiliations:** ^1^ Department of Microbiology, Harbin Medical University, Harbin 150081, China; ^2^ Department of Cell Biology, Harbin Medical University, Harbin 150081, China; ^3^ Department of Infectious Diseases, The Second Affiliated Hospital, Harbin Medical University, Harbin 150081, China

**Keywords:** AUF1, microRNA biogenesis, miR-122, Dicer1, hepatocellular carcinoma

## Abstract

Hepatocellular carcinoma (HCC) is one of the common cancers worldwide, especially in developing countries. Although the chronic infections of hepatitis B and C viruses have been established as the etiological factors of HCC, the mechanism for the tumorigenesis and development of HCC is still unclear. The liver-specific microRNA-122 (miR-122), an established tumor-suppressor miRNA, is often down-regulated in HCC, while the underlying mechanism is not well understood. Here we report that the AU-rich element-binding factor AUF1 suppresses the expression of Dicer1, the type III RNase that is required for microRNA maturation, leading to the inhibited biogenesis of miR-122. Overexpression of AUF1 led to the decreased expression of Dicer1 and miR-122, while the level of the miR-122 precursor (pre-miR-122) was increased. On the other hand, siRNA of AUF1 (siAUF1) increased the levels of Dicer1 mRNA and miR-122, but it reduced the abundance of pre-miR-122. Consistent with the reported data, this study demonstrated that AUF1 and Dicer1 showed opposite expression pattern in both human HCC tissues and cell lines. In addition, AUF1 inhibited the expression of Dicer1 by interacting with the 3′ untranslated region (3′UTR) and coding region of *DICER1* mRNA. Moreover, the knockdown of AUF1 by siRNA altered the expression of other miRNAs and promoted HCC cell death. In conclusion, AUF1 down-regulates the expression miR-122 by interacting with the 3′UTR and coding region of *DICER1* mRNA and suppressing Dicer1 expression. The AUF1/Dicer1/miR-122 pathway might play a critical role in the development of HCC.

## INTRODUCTION

Hepatocellular carcinoma (HCC) is one of the most common cancers in developing countries, and more than 55% of all HCC cases were diagnosed in China [1]. Despite of the development of advanced techniques for the diagnosis and treatment, the mortality of HCC is still rising [2]. Multiple risk factors, such as chronic infection of hepatitis B and C viruses (HBV, HCV), alcohol abuse contribute to development of HCC [3–5]. Although the molecular mechanism for the pathogenesis of HCC has not been well described, mounting evidence has demonstrated the aberrant activation of cell signaling pathways such as AKT/mTOR, RAS/MAPK, Wnt/β-catenin, insulin-like growth factor (IGF), and nuclear factor-κB (NF-κB) were involved in the development of HCC [6, 7]. In recent years, growing evidence has suggested that microRNAs (miRNAs) play a critical role in the progression, invasion, and angiogenesis of HCC [8–11].

miRNAs constitute a class of small endogenous non-coding RNAs that post-transcriptionally regulate gene expression [12]. The canonical biogenesis pathway of miRNA begins with the transcription of miRNA-coding gene to produce a stem-loop structured primary miRNA (pri-miRNA), which is cleaved by RNase III Drosha to generate a ∼60–70 nt long precursor, the pre-miRNA [13]. The pre-miRNA is then exported from the nucleus to the cytoplasm by exportin-5. In the cytoplasm, pre-miRNA is processed by RNase III Dicer1 into a ∼22 nt double-stranded RNA, miRNA-miRNA^*^ [14]. One strand in the duplex is the mature miRNA and the other complementary strand (miRNA^*^) becomes a passenger [14]. The mature miRNA is then incorporated into the RNA induced silencing complex (RISC) which is essentially constituted by the Argonaute (AGO) proteins [15, 16]. In metazoans, miRNA in the RISC regulates gene expression through targeting mRNA largely by incomplete base-pairing, resulting in the down-regulation of protein synthesis via the suppression of translation or the degradation of the target mRNA [16]. While miRNAs typically bind the 3′ untranslated region (3′UTR) of the target mRNA, the coding sequences and 5′UTR can also be the targets of miRNAs [17–20]. Because of miRNAs’ important role in physiological and pathological processes, the biogenesis of miRNAs is tightly regulated. Studies have shown that the expression of Dicer1, the RNase III endonuclease which processes the precursor miRNAs, can be suppressed by the AU-rich element RNA binding protein 1 (AUF1), also known as heterogeneous nuclear ribonucleoprotein D (HNRNPD) [21]. Moreover, AUF1 modulates the function of miRNAs through either influencing the recruitment of RISC to mRNA target site or directly binding miRNAs [22, 23].

miR-122 is the most abundant hepatic miRNA that constitutes 70% of miRNAs in the adult liver [24]. Increasing evidence has shown that miR-122 plays a central role in the development, differentiation, homeostasis, and functions of the liver [24]. miR-122 also directly interacts with the genomic RNA of HCV and promotes viral replication [25–27]. miR-122 is considered as tumor suppressor gene, because decreased miR-122 abundance is frequently found in HCC and it is often related with the invasion, metastasis, and poor prognosis of HCC [28–30]. So far, it remains unknown why the biogenesis of miR-122 is down-regulated in HCC.

Given the importance of miR-122 in liver diseases, the present study aims to determine the molecular basis underlying the reduced expression of miR-122 in HCC. Here we demonstrated that the increased expression of AUF1, which suppresses the expression of Dicer1, contributes to the down-regulated miR-122 in HCC.

## RESULTS

### The expression of AUF1 and Dicer1 in HCC tissues and cell lines

**Figure 1:**
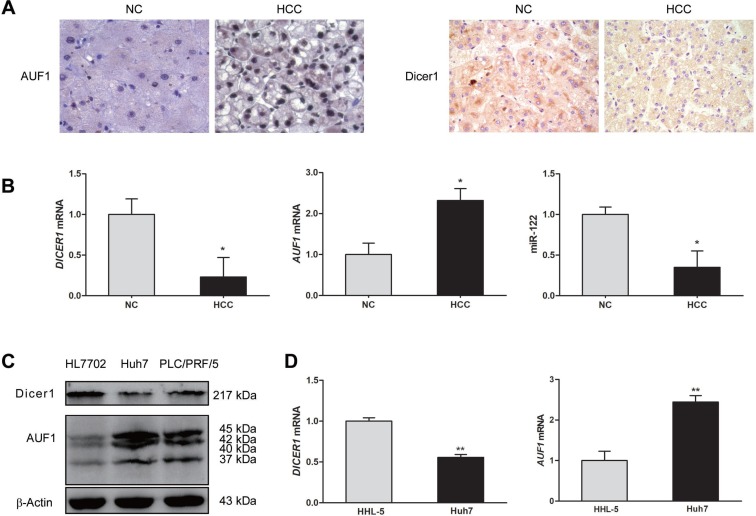
The expression of AUF1 and Dicer1 in HCC tissues and cell lines (**A**) Hepatocellular carcinoma (HCC) tissues and the adjacent non-tumor tissues (NC) from HCC patients were subjected to immunohistochemistry staining with anti-AUF1 and anti-Dicer1 antibodies (×400). (**B**) Total RNA was extracted from HCC tissues and adjacent non-tumor tissues. The relative levels of *DICER1* mRNA, *AUF1* mRNA, and miR-122 were determined by qRT-PCR compared to *GAPDH* mRNA. Data are represented as mean ± SD. *n* = 20; ^*^P < 0.05. (**C**) HL7702, Huh7, and PLC/PRF/5 cells were cultured in 6-well plate to 80% confluency. Cellular proteins were extracted, and the expression of Dicer1 and AUF1 was determined by Western blotting. AUF1 includes four isoforms (37, 40, 42, and 45 kDa). (**D)** HHL-5 and Huh7 cells were cultured in 6-well plate to 80% confluency. The level of *DICER1* mRNA and *AUF1* mRNA was determined by qRT-PCR normalized to *GAPDH* mRNA. Data are represented as mean ± SD. *n* = 4, ^**^P < 0.01.

We began this study by determining the expression of AUF1, Dicer1, and miR-122 in both HCC liver tissues and HCC cell lines. The expression of AUF1 and Dicer1 in HCC tissues and the adjacent non-tumor tissues from 20 patients were determined by immunohistochemistry and quantitative real-time PCR (qRT-PCR). The protein levels of AUF1 and Dicer1 in HCC cell lines HL7702, Huh7, and PLC/PRF/5 were determined by Western blotting. As shown in Figure 1, compare to non-tumor liver tissues, AUF1 expression was increased in HCC and it was located in both the nucleus and the cytoplasm of cancer cells (Figure 1A). Dicer1 was mainly located in the cytoplasm of the cells of non-tumor liver tissues, while weak expression of Dicer1 in the tissues of HCC was identified (Figure 1A). *AUF1* mRNA level was significantly increased in cancer tissues compared with that in non-tumor tissues, while *DICER1* mRNA level in HCC tissues was significantly decreased compared with that in non-tumor tissues (Figure 1B). Consistent with the previous findings [29], miR-122 was also found down-regulated in HCC tissues in this study (Figure 1B). In the case of HCC cell lines, Huh7 and PLC/PRF/5 cells expressed relatively high level of AUF1 and low level of Dicer1. In contrast, HL7702 cells expressed lower level of AUF1 and higher level of Dicer1 than the other two cell lines (Figure 1C). The level of *AUF1* mRNA in HHL-5 cells, a normal hepatocyte cell line, was lower than that in Huh7 cells, while the level *DICER1* mRNA in HHL-5 cells was higher than that in Huh7 cells (Figure 1D). These data suggest that AUF1, Dicer1, and miR-122 are present in HCC with altered expression profile.

### AUF1 suppresses the expression of Dicer1

**Figure 2:**
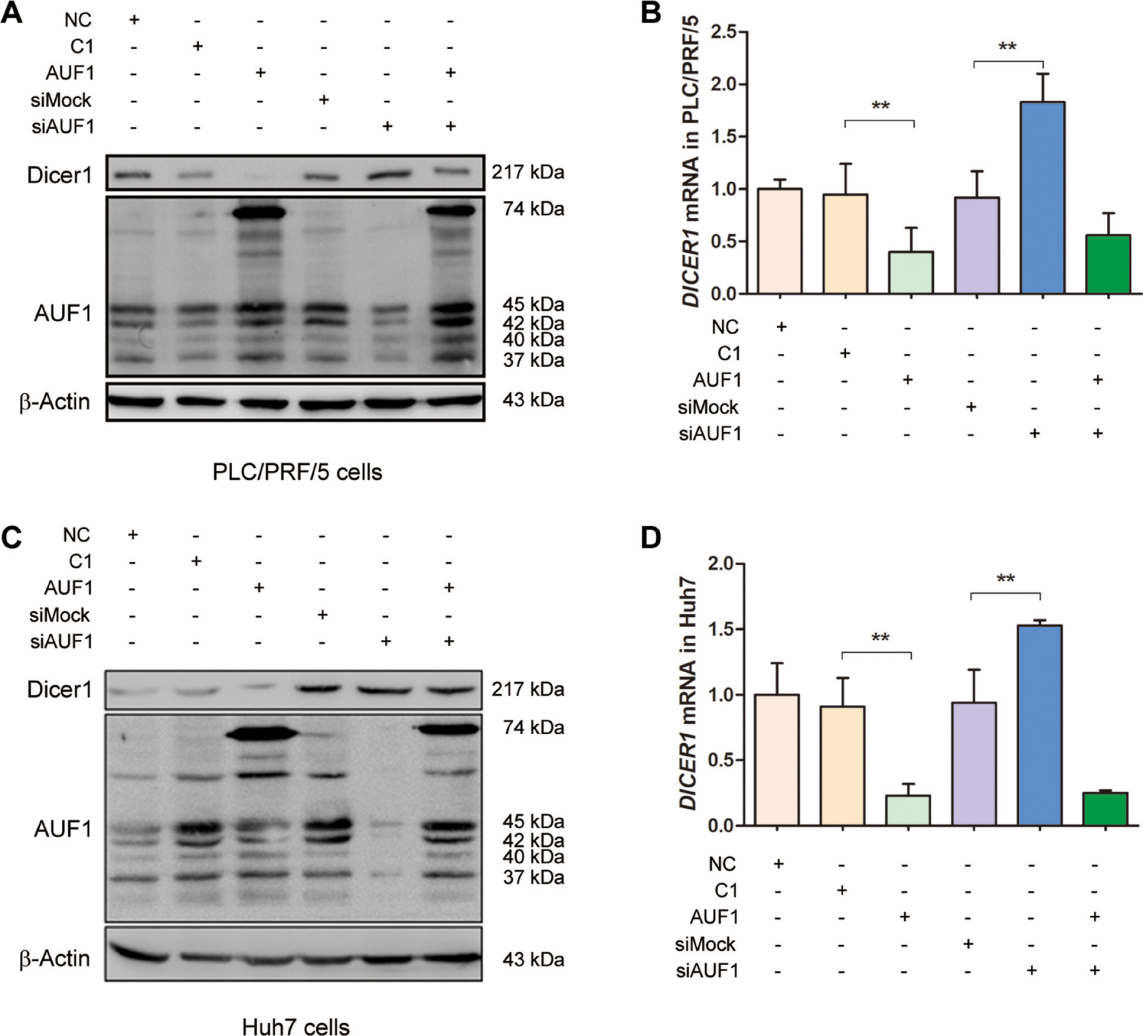
AUF1 suppresses the expression of Dicer1 (**A**, **C**) PLC/PRF/5 (A) and Huh7 cells (C) were grown in 6-well plate to 70% confluency. Cells were transfected with pEGFP-AUF1 (AUF1) or siRNA of AUF1 (siAUF1) for 48 h. Control cells were transfected with liposome (NC), pEGFP-C1 (C1), and mock siRNA (siMock), respectively. Proteins were extracted and subjected to Western blot analysis. (**B**, **D)** The level of *DICER1* mRNA was determined by qRT-PCR. Data are represented as mean ± SD. *n* = 4; ^**^P < 0.01. Experiments were repeated three times and representative results were presented.

To explore the association between RNA-binding protein AUF1 and endoribonuclease Dicer1, the expression of Dicer1 was studied by the overexpression of AUF1 or the inhibition of AUF1 with siRNA. PLC/PRF/5 and Huh7 cells were transfected with pEGFP-AUF1 or the small interference RNA of AUF1 (siAUF1) for 48 h. Total RNA was extracted and *DICER1* mRNA was detected by qRT-PCR. Cellular proteins were extracted and the expression of AUF1 and Dicer1 was determined by Western blotting. As shown in Figure 2, overexpression of AUF1 significantly down-regulated the level of *DICER1* mRNA (Figure 2B, 2D) and almost completely blocked the protein synthesis of Dicer1 (Figure 2A, 2C), while cells transfected with siAUF1 showed an increase on the protein level of Dicer1 (Figure 2A, 2C). The level of *DICER1* mRNA was also significantly increased in the cells transfected with siAUF1 (Figure 2B, 2D). However, the level of *DICER1* mRNA was down-regulated in both cell lines co-transfected with pEGFP-AUF1 and siAUF1 (Figure 2B, 2D), possibly due to the relative large amount of AUF1 mRNA generated endogenously and exogenously (transfected plasmid). Indeed, the AUF1 protein level in these cells was almost the same as that in cells with pEGFP-C1 and siMock controls (Figure 2A, 2C). These data suggest that AUF1 promotes the degradation of *DICER1* mRNA and suppresses the expression of Dicer1.

### AUF1 regulates the maturation of miR-122

**Figure 3:**
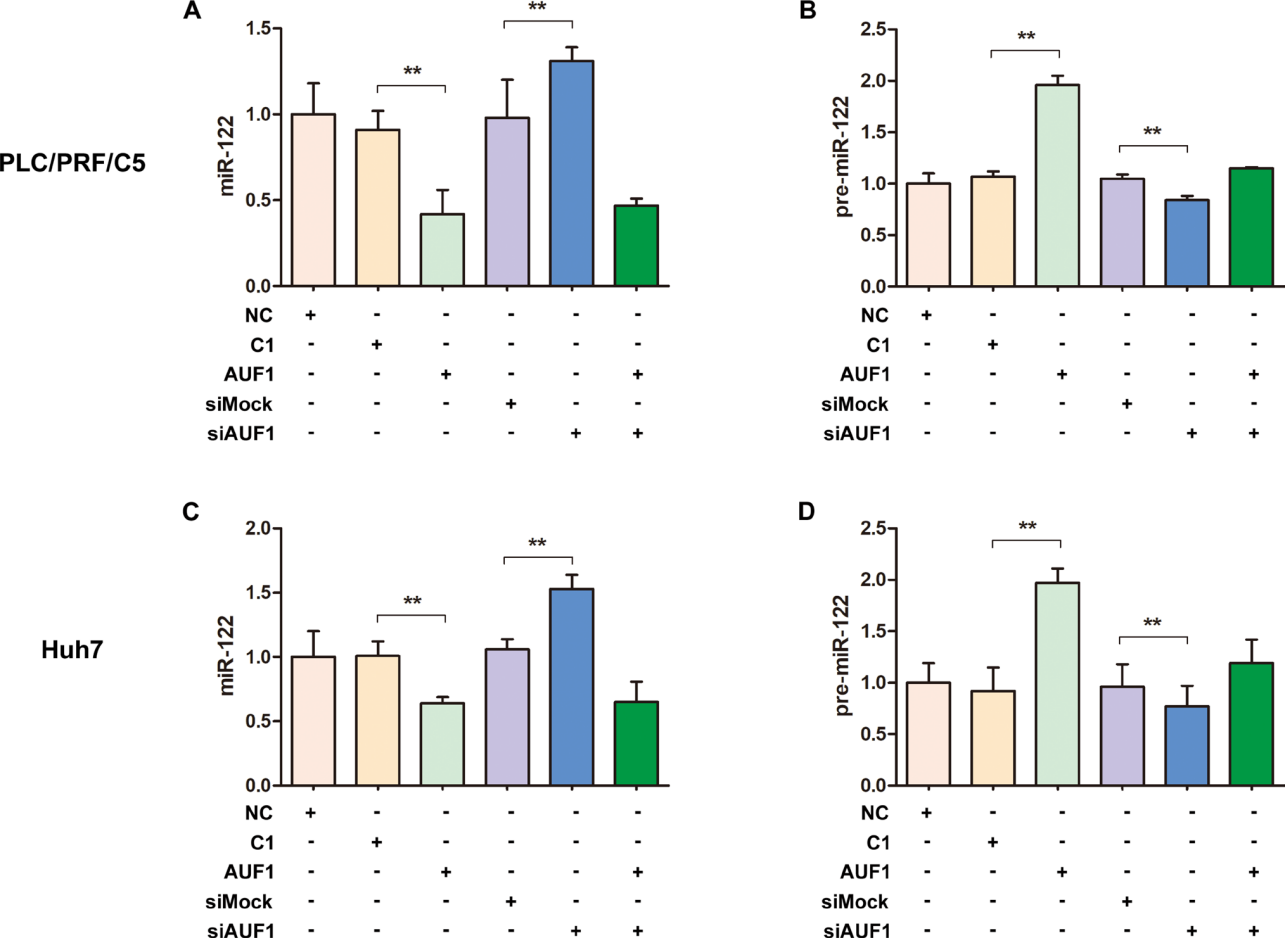
AUF1 regulates the maturation of miR-122 PLC/PRF/5 and Huh7 cells were transfected with pEGFP-AUF1 or siAUF1, or co-transfected with both pEGFP-AUF1 and siAUF1 for 48 h. The levels of miR-122 (**A**, **C**) and pre-miR-122 (**B**, **D**) were determined by qRT-PCR. Control cells were transfected with pEGFP-C1 (C1), mock siRNA (siMock) or liposome (NC). *n* = 4. ^**^P < 0.01.

Dicer1 is the RNase required for miRNA maturation. Our data indicate that Dicer1 expression can be regulated by AUF1, we further studied if AUF1 influences the maturation of miR-122, the most abundant miRNA in the liver. PLC/PRF/5 and Huh7 cells were transfected with pEGFP-AUF1 or siAUF1 for 48 h, and the levels of miR-122 and pre-miR-122 were determined by qRT-PCR. As shown in Figure 3, the abundance of miR-122 was reduced significantly in both PLC/PRF/5 and Huh7 cells overexpressing AUF1, while miR-122 level was increased markedly in both cell lines transfected with siRNA of AUF1 (Figure 3A, 3C). In contrast, the abundance of pre-miR-122 was increased dramatically in the cells overexpressing AUF1 and decreased in the cells transfected with siRNA of AUF1 (Figure 3B, 3D). Collectively, these data suggest that the maturation from pre-miR-122 to miR-122, a step mainly processed by Dicer1, is somehow blocked by AUF1.

### AUF1 regulates Dicer1 expression through interacting with the 3′UTR and coding region of *DICER1* mRNA

**Figure 4:**
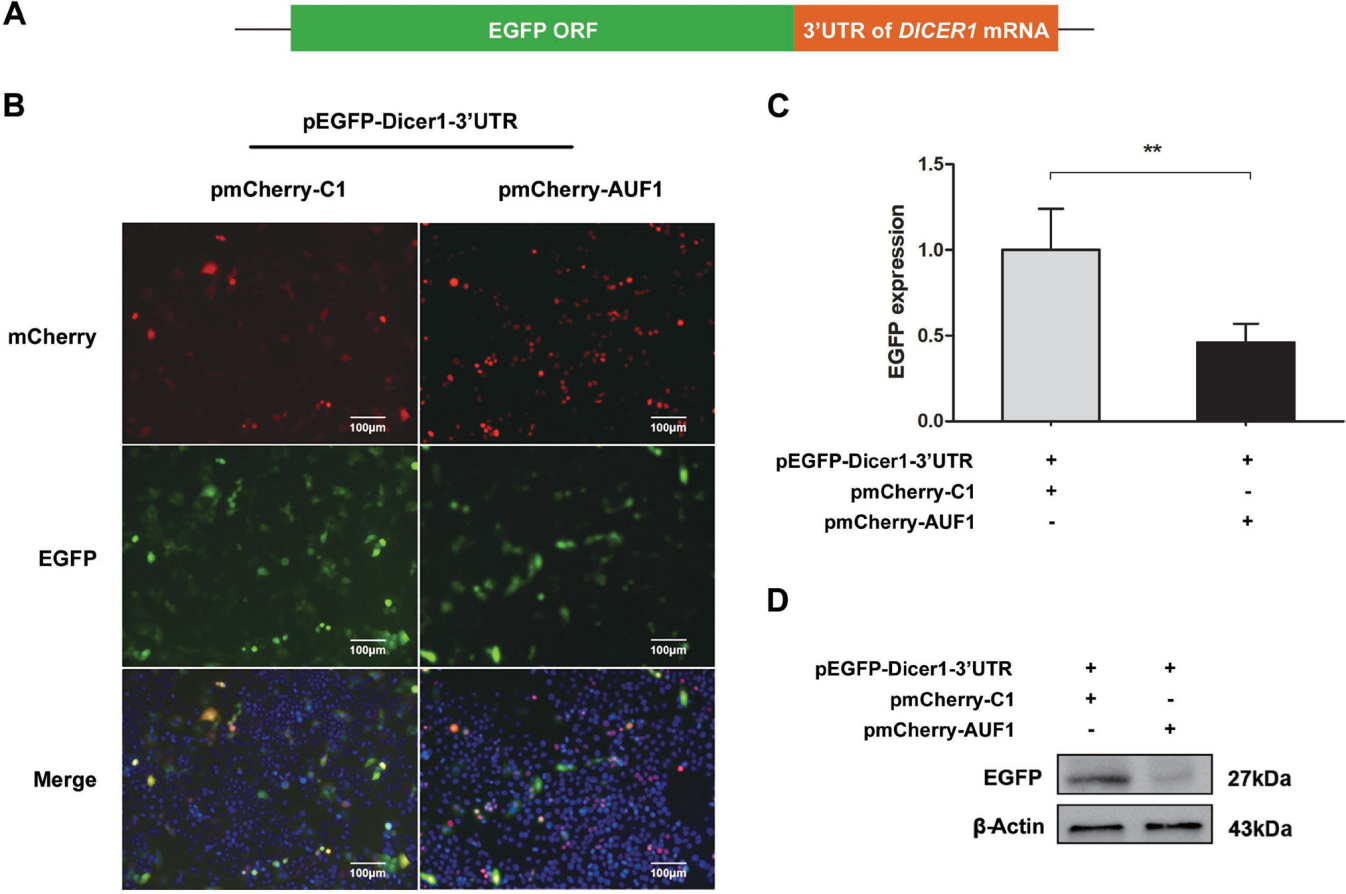
AUF1 interacts with the 3′UTR of *DICER1* mRNA and regulates its expression (**A**) The diagram of pEGFP-Dicer1-3′UTR. (**B**) Vero cells were co-transfected with pEGFP-Dicer1-3′UTR and pmCherry-AUF1 for 24 h, and the expression of EGFP and AUF1 was observed by fluorescence microscopy. (**C**) The fluorescence quantity was measured by fluorospectrometer. (**D**) The expression of EGFP protein in the treated Vero cells. *n* = 3. ^**^P < 0.01. Experiments were repeated three times and representative results were presented.

**Figure 5:**
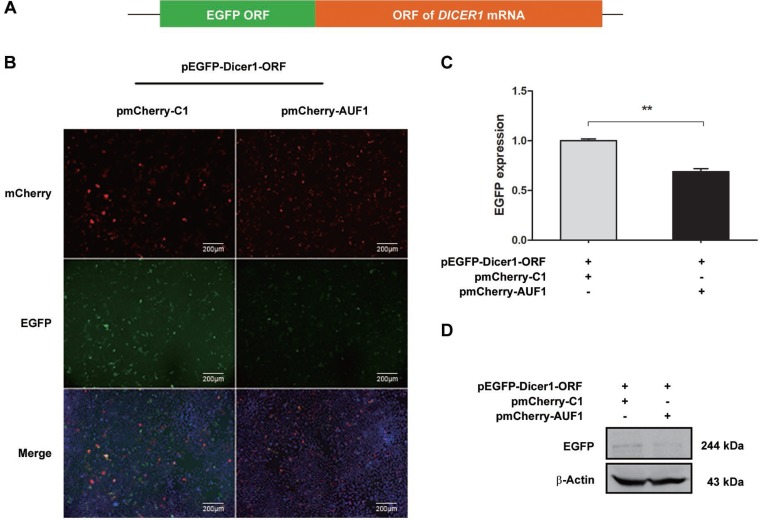
AUF1 interacts with the coding region of *DICER1* mRNA and regulates its expression (**A**) The diagram of pEGFP-Dicer1-ORF. (**B**) Vero cells were co-transfected with pEGFP-Dicer1-ORF and pmCherry-AUF1 for 24 h, and the expression of EGFP and AUF1 was observed by fluorescence microscopy. (**C**) The fluorescence quantity was measured by fluorospectrometer. (**D**) EGFP protein expression in the treated Vero cells. *n* = 3. ^**^P < 0.01. Experiment was repeated three times and representative results were presented.

In consistent with our results above, AUF1 has been demonstrated to repress the expression of Dicer1 [21]. To examine whether AUF1 regulates the expression of Dicer1 via interacting with *DICER1* mRNA, we constructed pEGFP-Dicer1-ORF and pEGFP-Dicer1-3′UTR, in which the 3′UTR or open-reading frame (ORF) of *DICER1* mRNA was inserted downstream of the EGFP sequence, respectively. Vero cells were co-transfected with pEGFP-Dicer1-3′UTR and pmCherry-AUF1 or with pEGFP-Dicer1-ORF and pmCherry-AUF1, and the expression of EGFP and AUF1 was observed by fluorescence microscopy and the fluorescence intensity was measured by a fluorospectrometer (Nanodrop 3300, Thermo). As shown in Figures 4 and 5, the intensity of EGFP fluorescence (Figure 4B, 4C) and the protein of EGFP (Figure 4D) were decreased significantly in the cells co-transfected with pEGFP-Dicer1-3′UTR and pmCherry-AUF1.

Similarly, in the cells co-transfected with pEGFP-Dicer1-ORF and pmCherry-AUF1, the intensity of EGFP fluorescence (Figure 5B, 5C) and the protein of EGFP (Figure 5D) were also decreased significantly. These data indicate that AUF1 inhibits Dicer1 expression through interacting with both the 3′UTR and ORF of *DICER1* mRNA.

### AUF1 affects the expression of oncogenic miRNAs in HCC cells

**Figure 6:**
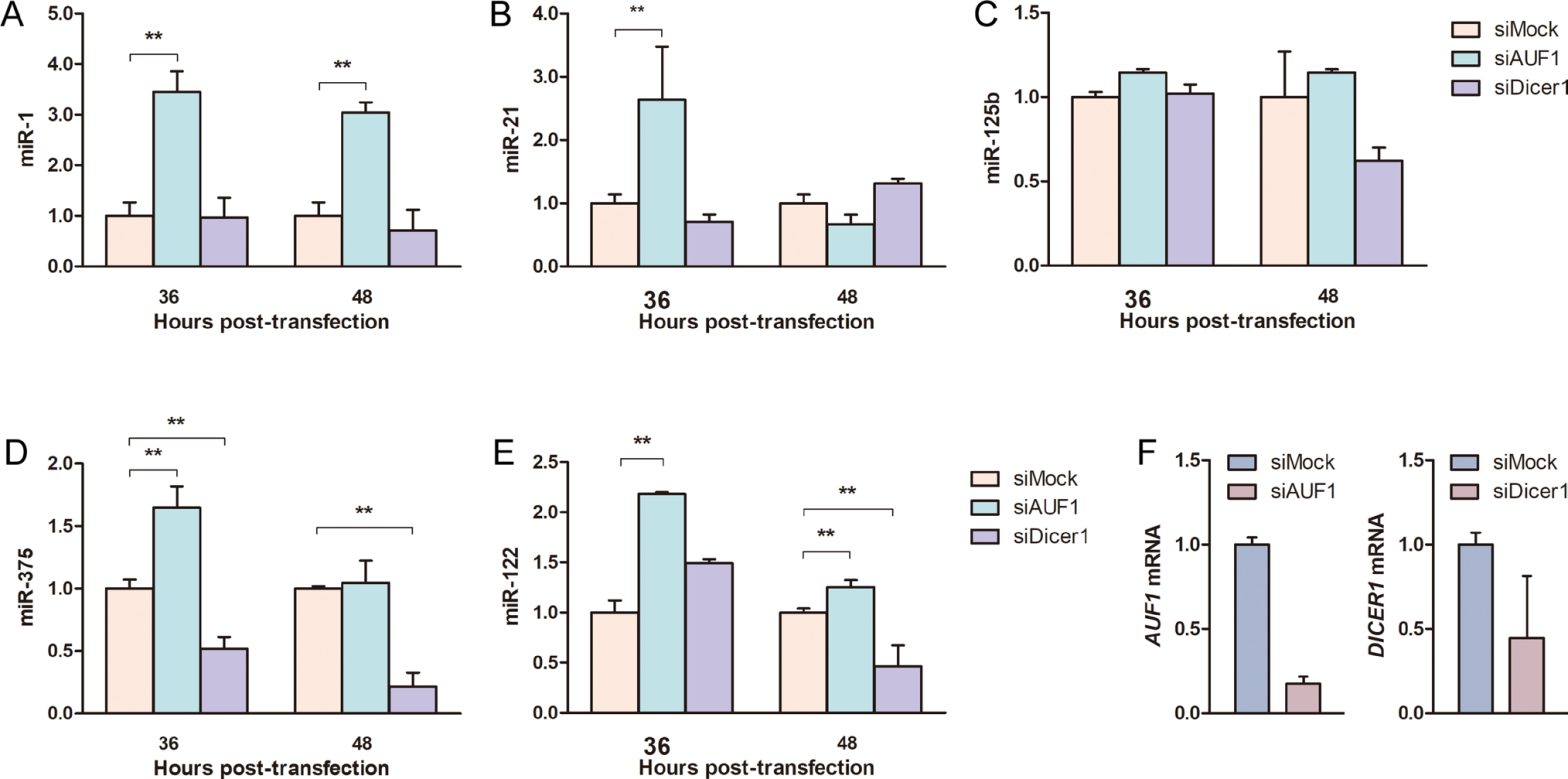
AUF1 affects oncogenic miRNA expression in hepatocellular carcinoma cells Huh7 cells were transfected with siAUF1 and siDicer1 for 36–48 h. (**A**–**E**) The levels of oncogenic miRNAs (miR-1, miR-21, miR-125b, miR-375) and miR-122 were determined by qRT-PCR. (**F**) The mRNAs of *AUF1* and *DICER1* were detected by qRT-PCR at 36 h post-transfection to show the knockdown efficiency of siRNAs. Control cells were transfected with siMock. The abundance of miRNAs and mRNAs was normalized to *GAPDH* mRNA. Data are presented as mean ± SD. *n* = 4. ^**^P < 0.01.

Based on above data that AUF1 can modulate the expression of miR-122 by targeting *DICER1* mRNA, we further evaluated whether other miRNAs could be affected by AUF1 in hepatoma cells. As shown in Figure 6, AUF1 and Dicer1 were knocked down by siAUF1 and siDicer1, respectively (Figure 6F). The levels of four oncogenic miRNAs (miR-1, miR-21, miR-125b, and miR-375) were determined by qRT-PCR. Similarly, AUF1 knockdown significantly increased the abundance of miR-1, miR-21, and miR-375, while Dicer1 knockdown reversed the upregulation of these miRNAs in Huh7 cells. Although miR-125b was not changed dramatically during knockdown treatment (Figure 6A–6E), these data suggest at least that aberrant AUF1 expression may globally disturb the expression profile of miRNAs in HCC cells, especially the oncogenic miRNAs.

### Inhibited expression of AUF1 promotes HCC cell death

**Figure 7:**
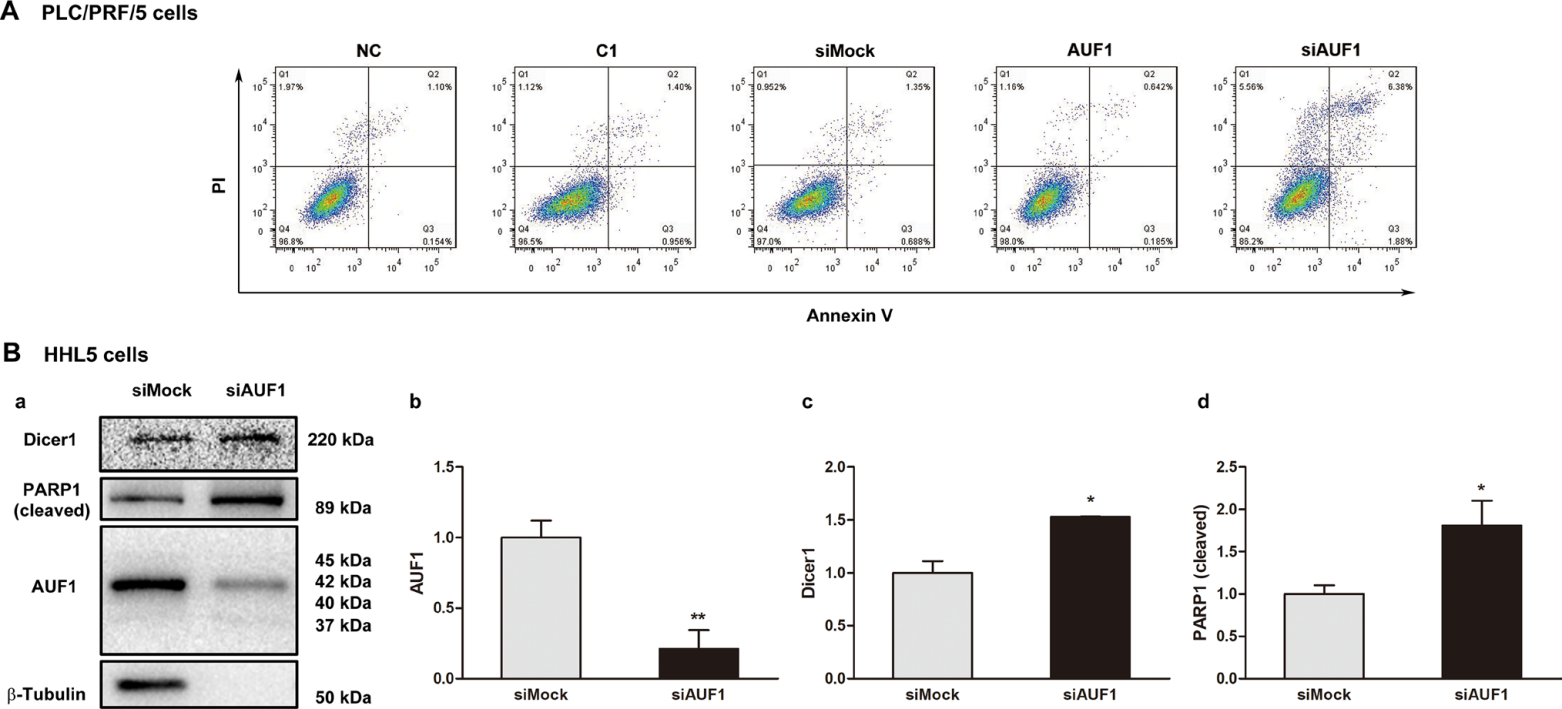
Inhibited expression of AUF1 promotes HCC cell death (**A**) PLC/PRF/5 cells were grown in 6-well plate. The cells were transfected with pmCherry-AUF1 (AUF1) or siRNA of AUF1 (siAUF1) for 24 h in the medium without serum. Apoptosis was determined by flow cytometry. Control cells were transfected with liposome (NC), pmCherry-C1 (C1) or siMock. (**B**) 80% confluent HHL-5 cells were transfected with siAUF1 or siMock for 24 h in the medium without serum. The expression of Dicer1, AUF1, and cleaved PARP1 was determined by Western blotting (a). The relative expression levels are presented as mean ± SD (b, c, d). *n* = 4, ^*^P < 0.05, ^**^P < 0.01.

miR-122 has been demonstrated as tumor suppressor [31]. Our previous study showed that miR-122 promotes the apoptosis of HCC cells [32]. Since the present study found that the increased expression of AUF1 reduced the abundance of miR-122 by blocking its maturation, apoptosis was further examined when AUF1 was overexpressed or inhibited by siRNA. As shown in Figure 7, the overexpression of AUF1 seems have little impact on the apoptosis of PLC/PRF/5 cells (Figure 7A), possibly due to the fact that PLC/PRF/5 cells express high level of endogenous AUF1. When cells transfected with siAUF1, increased number of cells in late phase of apoptosis (quadrant 2) or necrosis (quadrant 1) was observed, compared with that in the mock-transfected cells (Figure 7A). Accordingly, AUF1 knockdown in HHL-5 cells led to an increase of the cleaved poly (ADP-ribose) polymerase 1 (PARP1), suggesting the inhibitory effect of AUF1 on apoptosis (Figure 7B). Similar results were also observed in a xenograft mouse model engrafted with Huh7 cells and treated with siAUF1 (Supplementary Figure 1). These results demonstrate that the inhibited expression of AUF1 promotes HCC cell death.

## DISCUSSION

It has been demonstrated that the abundance of miR-122 is usually down-regulated in liver cancer [29], while the underlying mechanism is not understood. In this study, we evaluated the association between the RNA-binding protein AUF1 and miR-122. Our data suggest that AUF1 suppresses the maturation of miR-122 through inhibiting Dicer1 expression by interacting with the 3′UTR and ORF of *DICER1* mRNA.

We began this study by measuring the expression of AUF1, Dicer1, and miR-122 in both HCC cell lines and the tissues from HCC patients. In consistent with the previous data [24], miR-122 was significantly down-regulated in cancer tissues of HCC patients, compared to the matched non-cancerous liver tissues. The expression of AUF1 was markedly increased, while Dicer1 was significantly reduced in the cancer tissues of HCC patients. In the tested HCC cell lines, increased expression of AUF1 was observed in Huh7 and PLC/PRF/5 cells, compared with that in HL7702 cells. In contract, Dicer1 expression was relatively higher in HL7702 cells, compared with that in Huh7 and PLC/PRF/5 cells. To keep comparable to the results obtained from tissues of HCC patients, Huh7 or PLC/PRF/5 cells was used in the subsequent experiments. Although the number of tissue samples from HCC patients was relatively small, these data, in consistent with the previous observation [21], at least indicate that the overall expression profiles of AUF1 and Dicer1 were altered in HCC with increased level of AUF1 and decreased level of Dicer1.

As AU-rich element binding proteins, AUF1 plays an important role in the post-translational regulation of gene expression through controlling mRNA turnover [33]. The binding of AUF1 may result in the degradation or stabilization of the target mRNA, depending upon the sequence of the AU-rich elements (ARE) or cell type. To date, the limited data addressing the expression of AUF1 in HCC patients revealed that AUF1 was highly expressed in the cancer tissues compared with normal liver tissues [34, 35]. *In vivo* study using transgenic mice overexpressing p37^AUF1^ isoform resulted in the accumulation of mRNA of *FOS, MYC*, and *JUN* and tumorigenesis [36]. High level of c-fos was also found in HCC cell line with high metastasis potential [37]. Thus, our data suggest that the upregulated AUF1, at least in part, contributes to the uncontrolled proliferation of liver cells, possibly through targeting protooncogene *FOS*.

Consistent with our results, down-regulated expression of Dicer1 has been demonstrated in HCC tissues, compared with the adjacent non-tumor tissues. Importantly, the decreased expression of Dicer1 correlates with poor survival of HCC patients [38, 39]. In contrast, overexpression of Dicer1 inhibits the proliferation and promotes the apoptosis of HCC cells [38].

The association between AUF1 and Dicer1 has been demonstrated previously, in which AUF1 promoted the degradation of *DICER1* mRNA through binding to its 3’UTR and ORF regions [21]. By performing overexpression and siRNA inhibition of AUF1, we confirmed that Dicer1 expression was indeed inversely correlated with AUF1. The interaction between AUF1 and the 3’UTR or coding region of *DICER1* mRNA was also demonstrated in this study. Importantly, this study shows that AUF1 suppresses the biogenesis of miR-122 at the step from pre-miRNA to miRNA, possibly by promoting the degradation of *DICER1* mRNA. Our results suggest that the up-regulated expression of AUF1 and insufficient Dicer1 might be crucial for the dysregulated biogenesis of miRNAs including miR-122 in HCC.

Based on the available results, we postulated that the biogenesis of all miRNAs in HCC cells might be affected by the aberrant expression of AUF1 and, subsequently, Dicer1. To this end, four miRNAs (miR-1, miR-21, miR-125b, and miR-375) were selected to represent the impact of AUF1. Just as its impact on miR-122, AUF1 knockdown resulted in the decreased levels of miR-1, miR-21, and miR-375. It is noteworthy that miR-122, miR-1, miR-21, miR-125b, and miR-375 are so called onco-miRs, which play various roles in tumor development such as carcinogenesis, malignant transformation, and metastasis [40, 41].

**Figure 8:**
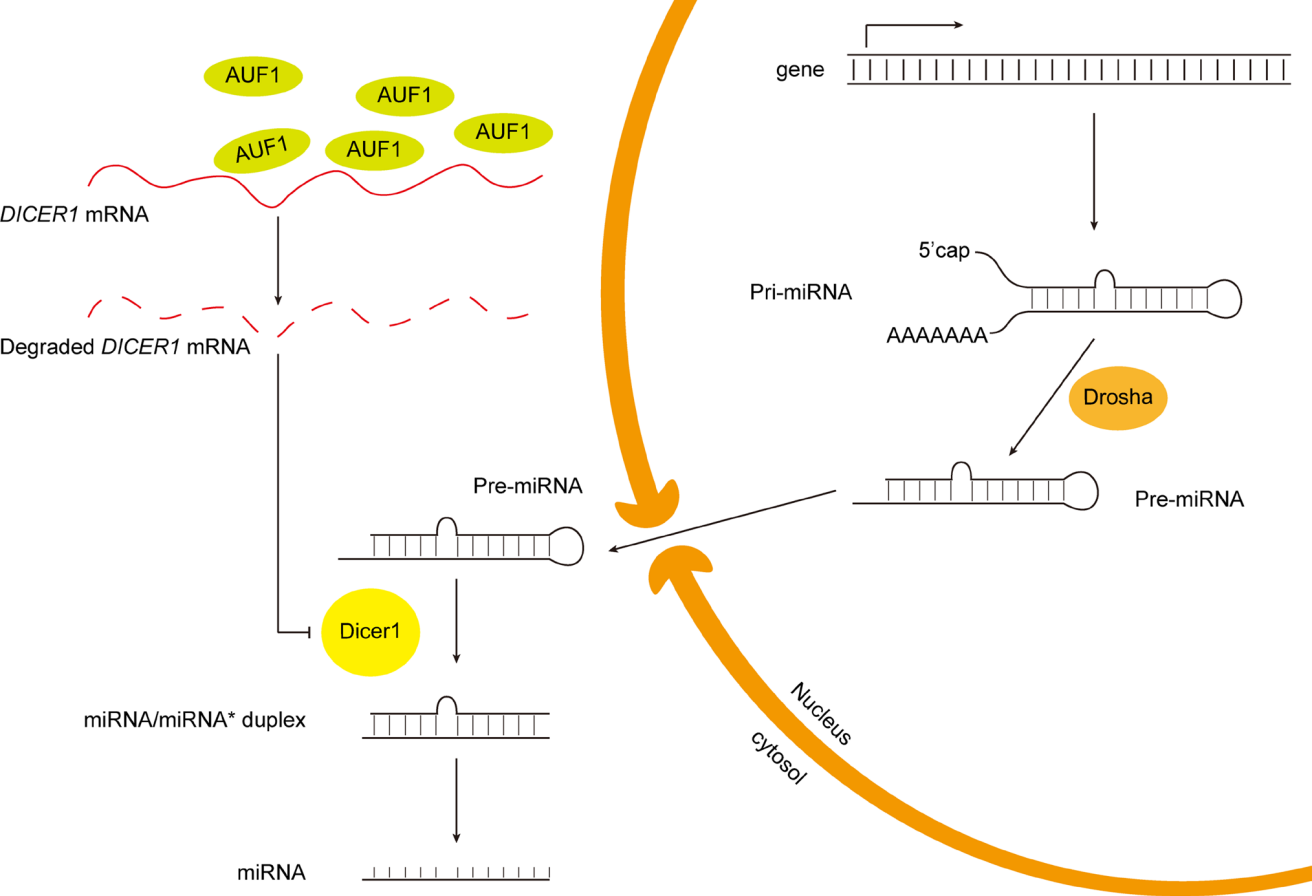
Schematic diagram of the putative mechanism of AUF1 regulating miR-122 maturation

The putative mechanism for the regulatory effect of AUF1 on miRNA expression through AUF1-Dicer1 interaction is summarized schematically in Figure 8. Given the large abundance and active biological role of miR-122 in liver, the change of miR-122 level might be a remarkable molecular event for liver cells. In contrast, the loss of other miRNAs may not be so influential as that of miR-122 due to their relative less abundance in either HCC or heathy liver cells.

In a previous study, we found that miR-122 promotes apoptosis and inhibits the viability of HCC cells [32]. Here we evaluated the effect of AUF1 on the apoptosis of HCC cells. Overexpression of AUF1 had no impact on apoptosis, possibly due to the fact that both cell lines, Huh7 and PLC/PRF/5, already express a high level of AUF1. Inhibited expression of AUF1 by siRNA promoted HCC cell death, which could be the result of the elevated level of miR-122. However, as an RNA-binding protein, AUF1 regulates the expression of a variety of proteins and non-coding RNAs [22]. Therefore, the effect of AUF1 on cell death observed in this study is definitely not conclusive, and further study is needed to verify this observation and the precise mechanism in which AUF1 exerts on cell death.

The present study indicates that AUF1 play a critical role in HCC. AUF1 is encoded by a single-copied gene localized in chromosome 4 of the human genome. Four AUF1 molecules in distinct molecular weight, p37^AUF1^, p40^AUF1^, p42^AUF1^, and p45^AUF1^, are generated by alternative splicing during transcription [22]. AUF1 affects expression through binding and directing the decay of its target mRNAs [22, 33]. This RNA-binding protein regulates the expression of a rich array of proteins, many of which are the key players in cell cycle, apoptosis, and inflammation [42, 43]. Studies have demonstrated that AUF1 shows both pro- and anti-apoptotic activity [35, 44, 45]. In this study, although we did not observe increased ratio of cells in the early phase of apoptosis when the expression of AUF1 was knocked down by specific siRNA, the proportion of cells in late apoptosis and necrosis was indeed elevated, indicating that AUF1 might play a role in the survival of HCC cells.

In summary, this study demonstrated that AU-rich element binding protein AUF1, which was found upregulated in the cancerous tissues of HCC patients, suppresses the maturation of miR-122 by interacting with the 3′UTR and coding region of *DICER1* mRNA, leading to the reduced expression of Dicer1. AUF1 knockdown by siRNA promotes the HCC cell death. Our results show that AUF1 may play crucial roles in the development of HCC.

## MATERIALS AND METHODS

### Clinical specimens

Surgically resected liver tissues from 20 patients with HCC were collected. Tumor and the surrounding non-tumor tissues were stored in pairs. Informed consent concerning the investigation was provided. The collection and usage of the clinical specimens were in accordance with the principles of the Declaration of Helsinki and approved by the Ethics Committee of Harbin Medial University.

### Cell culture

Human HCC cell lines Huh7, PLC/PRF/5, HL7702 and non-transformed human hepatocyte-derived cell line HHL-5 were obtained from the Type Culture Collection of the Chinese Academy of Sciences (Shanghai, China). Vero cells were maintained in the Department of Microbiology, Harbin Medical University, Harbin, China. Cells were cultured in Dulbecco’s modified Eagle’s medium (DMEM) (Invitrogen, Carlsbad, CA, USA) supplemented with 10% fetal calf serum (FCS) (Biological Industries, Israel) and 1% penicillin and streptomycin at 37° C with 5% CO_2_.

### Plasmid construction

To construct AUF1-expressing plasmids, pEGFP-AUF1, the sequence of the four AUF1 variants (p37^AUF1^, GenBank accession No. NM_031370; p40 ^AUF1^, GenBank accession No. NM_031369; p42^AUF1^, GenBank accession No. NM_002138; p45 ^AUF1^, GenBank accession No. NM_001003810) was cloned into pEGFP-C1, which was constructed previously [46]. Briefly, total RNA was extracted from HeLa cells and reverse transcription was carried out. To amplify AUF1, the forward primer 5′-TATAGCAAGCTTTCGGAGGAGCAGTTCGGCGGGG-3′ and reverse primer 5′-CGGCATGGTACCTTAGTATGGTTTGTAGCTATTTTGATGAC -3′ were used in PCR. The underlined sequences refer to the cleavage sites of *Hind* III and *Kpn* I, respectively. The PCR product was cloned into pEGFP-C1 between the sites of *Hind* III and *Kpn* I. The constructed pEGFP-AUF1 was verified by sequencing.

pmCherry-AUF1 was constructed by inserting the sequence of p45^AUF1^ into pmCherry-C1 (Clontech, Mountain View, CA). Briefly, p45^AUF1^ was amplified by PCR from pEGFP-AUF1 (p45 ^AUF1^) constructed above with the forward primer 5′-TATATAAAGCTTCGGGTGGAGGCGGTTCAGGCGGAGGTGGCTCTGGCGGTGGCGGATCGTCGGAGGAGCAGTTC-3′ and reverse primer 5′-GCGCG CGGTACCTTAGTATGGTTTGTAGCTATTTTG-3′. The underlined sequences refer to the cleavage sites of *Hind* III and *Kpn* I, respectively. The PCR product and pmCherry-C1 were digested by *Hind* III and *Kpn* I, and ligation reaction was performed. The obtained construct was verified by sequencing.

pEGFP-Dicer1-ORF was constructed by inserting the ORF of *DICER1* into pEGFP-C1 downstream of the EGFP sequence. In brief, the ORF of Dicer1 was amplified by PCR from pFRT/TO/FLAG/HA-DEST DICER (Addgene plasmid # 19881) [47] with the forward primer 5′-ATATATAAGCTTAAAAGCCCTGCTTT GCAACCCCTCAGCATGG-3′ and reverse primer 5′-ATATATGGGCCCTCAGCT ATTGGGAACCTGAGGTTGATTA-3′. The underlined sequences represent the cleavage sites of *Hind* III and *Apa* I (TaKaRa, Dalian, China), respectively. The PCR product was inserted into pEGFP-C1, and the obtained construct was verified by sequencing.

pEGFP-Dicer1-3′UTR was constructed by inserting the 3′UTR sequence of *DICER1* mRNA into pEGFP-C1 downstream of the EGFP sequence. Briefly, the 3′UTR sequence of *DICER1* mRNA was amplified by PCR from plasmid pIS1 DICER1 long UTR (Addgene, Cambridge, MA) with the forward primer 5′-ATATATGGATCCAACCGCTTTTTAAAATTCAAAACAA-3′ and reverse primer 5′-GCGCTAGGGCCCTTTTTTTTTTTTTTTTTTTTTTTTTTTTTTTTTTTT TTTTTTTTTTTGAACAGACGAT-3′. The underlined sequences represent the cleavage sites of the endonuclease *Bam*H I and *Apa* I, respectively. The PCR product was inserted into pEGFP-C1, and the obtained construct was verified by sequencing.

### Transfection

Cells were plated in 24-well culture plates at the density of 1 × 10^4^ cells/well and cultured in 300 μl serum-free medium for 24 h to 80% confluence. Transfection was performed with Lipofectamine 2000 (Invitrogen) according to the protocol recommended by the manufacturer. Briefly, 25 µl Opti-MEM medium (Invitrogen) was used to dilute 1.0 μl liposome and 0.5 μg plasmid or 0.27 μg siRNA (Genechem, Shanghai, China), and equal volume of the plasmid or siRNA and liposome was mixed at room temperature for 15 min. Cells were transfected by adding 100 μl of the Opti-MEM medium containing liposome and plasmid or siRNA at 37° C for 6 h, and then cells were grown at DMEM containg 10% FCS.

### RNA extraction and reverse transcription

Cells were cultured in 24-well plates to 80% confluence. Total RNA was extracted with TRIzol (Invitrogen) according to the protocol recommended by the provider. The quality of the extracted RNA was analyzed and RNA was stored at –80° C. RNAs of AUF1, Dicer1, and pre-miR-122 was reverse-transcribed by oligo(dT) or specific primers using the reverse-transcription kit (TaKaRa). The primers for the reverse transcription and amplification of miRNAs (miR-122, miR-1, miR-21, miR-125b, and miR-375) are listed in the Supplementary Table 1. The total reaction volume for reverse transcription was 10 μl in which 1 μl of reverse primer, 1 μl of RNA template, 2 μl of 5 × PrimeScript buffer, 5.5 μl of DEPC-treated ddH_2_O, and 0.5 μl of PrimeScript RT Enzyme Mix I (TaKaRa) were included. Reverse transcription was carried out for 15 min at 37° C and followed by the incubation at 85° C for 5 s by using DNA Engine Peltier Thermal Cycler (Bio-Rad, Hercules, CA, USA).

### Quantitative real-time PCR

The quantitative real-time PCR (qRT-PCR) was performed by using Lightcycler 2.0 (Roche, USA) according to the protocol recommended by the manufacturer of SYBR PrimeScript RT-PCR Kit II (TaKaRa). Reaction was carried out in the total volume of 20 μl of PCR mixture, which includes 10 μl of SYBR Premix Ex Taq II (TaKaRa), 0.8 μl of both forward and reverse primers (10 μM), 2 μl of cDNA template, and 6.4 μl of ddH_2_O. U6 and GAPDH served as the internal control for the quantification of miR-122 and mRNAs, respectively. Forty reaction cycles of PCR were carried out according to the following condition: 95° C 5 s, 55° C 20 s, 72° C 15 s. PCR product was calculated according to the 2^–ΔΔCt^ method described previously [32]. The primer sequences used for qRT-PCR were provided in the supplementary material (Supplementary Table 1).

### Western blot

Cell culture was washed with PBS and incubated with RIPA buffer [10 mM Tris–HCl (pH7.4), 150 mM NaCl, 1% NP-40, 1 mM EDTA, 0.1% SDS and 1 mM dithiothreitol] (Thermo, Waltham, MA) containing protease inhibitor PMSF (Beyotime, Shanghai, China) at ice for 30 min. Cell lysate was centrifuged in 12,000 × g at 4° C for 10 min. The protein extract was separated by SDS-PAGE and electro-transformed to polyvinylidene fluoride (PVDF) membranes (Millipore, Billerica, MA). The PVDF membranes were blocked at room temperature with 5% nonfat milk in TBS [10 mmol/L Tris-HCl (pH7.5), 0.5 mol/L NaCl, and 0.1% (v/v) Tween 20] buffer for 4 h, followed by the incubation with the primary antibodies against AUF1 (Millipore), Dicer1 (Millipore), and β-actin (Golden Bridge, Beijing, China) at 4° C overnight. After being washed with Tris-Buffered Saline with Tween-20 [TBST; 20 mM Tris–Cl (pH7.5), 0.5 mol/L NaCl, 0.05 % (v/v) Tween 20] for three times, the membranes were incubated with the horseradish peroxidase (HRP)-conjugated secondary antibodies at 37° C for 1 h. SuperSignal West Pico chemiluminescent substrate kit (Thermo) was used to visualize the blotting results. The blots were imaged with FluorChem R system (ProteinSimple, San Jose, CA, USA).

### Flow cytometry

Cells collected from cell culture were suspended in absolute ethanol. 500 µl of cellular suspension (containing 5 × 10^6^ cells) was incubated with 5 µl of annexin V-FITC and 5 µl of propidium iodide (PI) at dark for 15 min. Flow cytometry was performed by FACSort Flow Cytometer (BD Biosciences, San Jose, CA, USA) within 1 h after the labeling with fluorescence dyes. Data were acquired and analyzed by using the CELLQuest software (BD Lifesciences).

### Fluorescence microscopy

Cells grown on glass cover slips were co-transfected with pmChery-AUF1 and pEGFP-DICER1-3′UTR or with pmCherry-AUF1 and pEGFP-DICER1-ORF for 24 h. After being washed with PBS, the cells were treated with DAPI for 15 min at room temperature and washed again with PBS three times. Fluorescence images were captured with Axiovert 200 microscope equipped with a CCD camera controlled with ZEN software (Carl Zeiss, Gottingen, Germany).

### Immunohistochemistry

Immunohistochemistry staining was carried out according to the procedure recommended by the antibody providers. Briefly, human liver tissues collected from surgery were fixed and embedded with paraffin. Sections were prepared and subjected to antigen retrieval. After blocking the activity of endogenous peroxidase, the sections were incubated with the primary antibodies against AUF1 (Millipore) and Dicer1 (Millipore) at 1:2000 dilution, followed by the incubation with the secondary antibodies conjugated with horseradish peroxidase (HRP) (Golden Bridge, Beijing, China). Images were captured by Eclipse Ts100 microscope armed with a color CCD (Nikon).

### Statistical analysis

Data were presented as mean ± standard deviation (SD) or mean ± standard error of the mean (SEM) and analyzed by the Student’s *t* test using GraphPad Prism 5.0. *P* < 0.05 was considered statistically significant.

## SUPPLEMENTARY MATERIALS FIGURE AND TABLE



## Abbreviations

AUF1: AU-rich element binding protein
3′UTR: 3′ untranslated region
ORF: open-reading frame
HCC: hepatocellular carcinoma
miRNA: microRNA
HBV: hepatitis B virus
HCV: hepatitis C virus
