# Supplementary material for: RNA-binding protein AUF1 suppresses miR-122 biogenesis by down-regulating Dicer1 in hepatocellular carcinoma
Source: Oncotarget. 2018 Jan 9;9(19):14815–27. doi: 10.18632/oncotarget.24079 (PMC5871080; doi:10.18632/oncotarget.24079)
Supplement: Supplementary file 1 [file oncotarget-09-14815-s001.pdf]

RNA-binding protein AUF1 suppresses miR-122 biogenesis by down-regulating Dicer1 in hepatocellular carcinoma

SUPPLEMENTARY MATERIALS

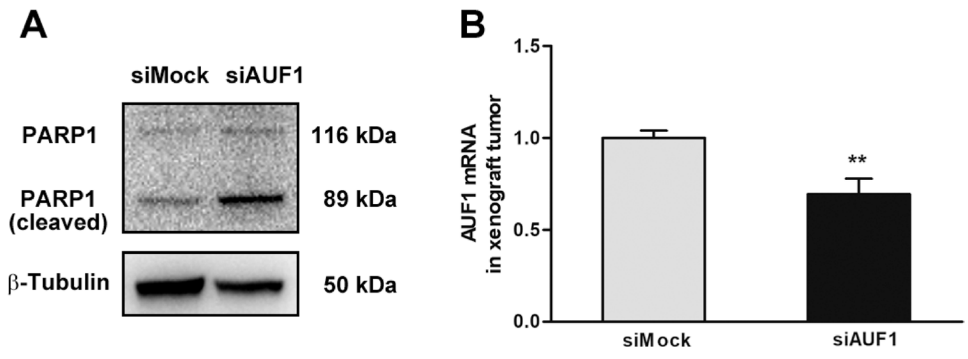

Supplementary Figure 1: AUF1 knockdown promotes the apoptosis of Huh7 xenograft tumor. Huh7 cells were transfected with siMock and siAUF1, respectively. About 10^5 transfected cells were inoculated subcutaneously to the back of BALB/c nude mice. The mice were sacrificed a week later and the proteins and RNAs of the xenografted tumors were extracted for Western blotting (PARP1) (**A**) and qRT-PCR (*AUF1* mRNA) (**B**). Data were presented as mean ± SD. $n = 4$, $^{**}P < 0.01$.

Supplementary Table 1: Primers used for reverse transcription and qRT-PCR

Primers	Sequences
U6 (reverse transcription)	CGCTTCACGAATTTGCGTGTCAT
U6 (qRT-PCR, sense)	GCTTCGGCAGCACATATACTAAAAT
U6 (qRT-PCR, antisense)	GCTTCGGCAGCACATATACTAAAAT
miR-122 (reverse transcription)	GTCGTATCCAGTGCGTGTCGTGGAGTCGGCAATTGCACTGGATACGACC AAACAC
miR-122 (qRT-PCR, sense)	GGGGTGGAGTGTGACAATG
miR-122 (qRT-PCR, antisense)	CAGTGCGTGTCGTGGAGT
GAPDH (qRT-PCR, sense)	ATGTTCCAATATGATTCCACCC
GAPDH (qRT-PCR, antisense)	CTGTAGCCAAATTCGTTGTCATAC
AUF1 (qRT-PCR, sense)	AGTGTAGATAAGGTCATGGATC
AUF1 (qRT-PCR, antisense)	CTCTTCCACGAGCTCTTC
Dicer1 (qRT-PCR, sense)	AGTGTAGATAAGGTCATGGATC
Dicer1 (qRT-PCR, antisense)	AATGGAAGCAGTTAGTCC
pre-miR-122 (qRT-PCR, sense)	CCTTAGCAGAGCTGTGGAGTGT
pre-miR-122 (qRT-PCR, antisense)	GCCTAGCAGTAGCTATTTAGTGTG
miR-1 (reverse transcription)	GGCTGCCGACCGTGTCGTGGAGTCGGCAATTGGTCGGCAGCCATACACAC
miR-1 (qRT-PCR, sense)	CTGTCACTCGAGCTGCTGGAATG
miR-1 (qRT-PCR, antisense)	ACCGTGTCGTGGAGTCGGCAATT
miR-21 (reverse transcription)	GTCGTATCCAGTCAGGGTCCGAGGTATTCGCACTGGATACGACTCAACA
miR-21 (qRT-PCR, sense)	GCCGCTAGCTTATCAGACTGATGT
miR-21 (qRT-PCR, antisense)	GTGCAGGGTCCGAGGT
miR-125b (reverse transcription)	CTCAACTGGTGTCGTGGAGTCGGCAATTCAGTTGAGTCACAAGT
miR-125b (qRT-PCR, sense)	ACACTCCAGCTGGGTCCCTGAGACCCTAAC
miR-125b (qRT-PCR, antisense)	GTGTCGTGGAGTCGGCAATTC
miR-375 (reverse transcription)	GTCGTATCCAGTGCAGGGTCCGAGGTATTCGCACTGGATACGACTCACGC
miR-375 (qRT-PCR, sense)	AGCCGTTTGTTCGTTCGGCT
miR-375 (qRT-PCR, antisense)	GTGCAGGGTCCGAGGT
